# Genotyping of *Burkholderia mallei* from an Outbreak of Glanders in Bahrain Suggests Multiple Introduction Events

**DOI:** 10.1371/journal.pntd.0003195

**Published:** 2014-09-25

**Authors:** Holger C. Scholz, Talima Pearson, Heidie Hornstra, Michaela Projahn, Rahime Terzioglu, Renate Wernery, Enrico Georgi, Julia M. Riehm, David M. Wagner, Paul S. Keim, Marina Joseph, Bobby Johnson, Joerg Kinne, Shanti Jose, Crystal M. Hepp, Angela Witte, Ulrich Wernery

**Affiliations:** 1 Department of Bacteriology and Toxinology, Bundeswehr Institute of Microbiology, German Center for Infection Research (DZIF), Munich, Germany; 2 Center for Microbial Genetics and Genomics, Northern Arizona University, Flagstaff, Arizona, United States of America; 3 Central Veterinary Research Laboratory, Dubai, United Arab Emirates; 4 Department of Microbiology, Immunbiology and Genetics, MFPL laboratories, University of Vienna, Vienna, Austria; University of California, San Diego, School of Medicine, United States of America

## Abstract

**Background:**

Glanders, caused by the gram-negative bacterium *Burkholderia mallei*, is a highly infectious zoonotic disease of solipeds causing severe disease in animals and men. Although eradicated from many Western countries, it recently emerged in Asia, the Middle-East, Africa, and South America. Due to its rareness, little is known about outbreak dynamics of the disease and its epidemiology.

**Methodology/Principal Findings:**

We investigated a recent outbreak of glanders in Bahrain by applying high resolution genotyping (multiple locus variable number of tandem repeats, MLVA) and comparative whole genome sequencing to *B. mallei* isolated from infected horses and a camel. These results were compared to samples obtained from an outbreak in the United Arab Emirates in 2004, and further placed into a broader phylogeographic context based on previously published *B. mallei* data. The samples from the outbreak in Bahrain separated into two distinct clusters, suggesting a complex epidemiological background and evidence for the involvement of multiple *B. mallei* strains. Additionally, the samples from Bahrain were more closely related to *B. mallei* isolated from horses in the United Arab Emirates in 2004 than other *B. mallei* which is suggestive of repeated importation to the region from similar geographic sources.

**Conclusion/Significance:**

High-resolution genotyping and comparative whole genome analysis revealed the same phylogenetic patterns among our samples. The close relationship of the Dubai/UAE *B. mallei* populations to each other may be indicative of a similar geographic origin that has yet to be identified for the infecting strains. The recent emergence of glanders in combination with worldwide horse trading might pose a new risk for human infections.

## Author Summary

Glanders is a disease of antiquity, recognized as a malady of equines by Hippocrates and Aristotle. The causative agent, *Burkholderia mallei*, is currently feared as a potential biological weapon and has been used as such in the American Civil War and both World Wars to cripple equine military components. In the more economically developed countries, glanders has been eradicated through large scale culling. As a result, our understanding of transmission dynamics and networks is limited. However, regions of endemicity still exist in Asia, the Middle-East, Africa, and South America where it infects solipeds and camels. These areas provide reservoirs for re-introduction of glanders into countries previously listed as glanders-free. Here, we demonstrate the utility of high-resolution genotyping and whole genome sequence analysis in the investigation of a recent outbreak of glanders in horses and camels in Bahrain, a previously declared glanders-free country. Our analyses demonstrate that not one, but two strains likely caused this outbreak, and that these strains probably came from a similar geographic region via importation of infected animals. Even with careful monitoring, the global trade of animals from glanders-endemic regions can re-introduce and possibly re-establish this disease in animal populations of countries that have previously eradicated it.

## Introduction

Glanders is a life-threatening, notifiable zoonotic disease which is fatal to both animals and humans. It is caused by the gram-negative bacterium *Burkholderia mallei* [1]. The only known reservoirs of *B. mallei* are solipeds, particularly horses. Chronically infected horses can be asymptomatic but may remain highly infectious.

As a highly infectious agent that can be transmitted by aerosol, causing invasive fatal disease in combination with resistance to multiple antibiotics, *B. mallei* is listed as a category B bio-threat agent by the CDC (www.bt.cdc.gov/agent/agentlist-category.asp). Licensed vaccines against the disease do not exist. Antibiotic treatment is cumbersome and requires the combination of at least two different antibiotics over several weeks [2].

Throughout the western world, glanders has been eradicated through large scale culling of infected animals. In developing countries, however, economic and cultural circumstances may hinder culling of asymptomatic animals and enable the persistence of glanders.

In recent years, several outbreaks of glanders occurred in the horse populations in Asia, Middle-East (Afghanistan, Kuwait, Iran, Iraq, Pakistan, Syria), Africa, and South America (Brazil) [3], [4]. Because of the recent rise in cases in multiple countries, glanders has regained the status of a re-emerging disease [5], [6].

Officially, Bahrain was free of glanders until an outbreak in the north (Jannusan, Shakhurah and Saar municipalities) that began in April 2010. Horses imported from Syria via Kuwait were suspected of introducing glanders and all equines in the area were quarantined and tested. By September 2010, the outbreak was considered to be resolved. However, in January 2011 the disease reoccurred in the same region of the country. Details on the outbreak are provided by the OIE (http://www.oie.int/wahis_2/public/wahid.php/Wahidhome/Home).

More than 6,700 horses and 250 donkeys (100% of the equine population in this region, representing about 80% of the total horse and donkey population in Bahrain) as well as three camels presenting with clinical symptoms were screened for glanders at OIE Reference Laboratory, the Central Veterinary Research Laboratory in Dubai, United Arab Emirates using prescribed Complement Fixation Testing (CFT) and an in-house cELISA [7]. In these investigations, 50 horses and one camel tested positive. *B. mallei* was isolated from eight horses and the single, positive camel.

Using *B. mallei*-specific real-time PCR and high resolution MLVA typing, we showed recently that the strain from the camel was genetically closely related to *B. mallei* strain Dubai 7 that was isolated from a horse during the contained outbreak of glanders in a quarantine station in the United Arab Emirates in 2004 [8]. In this current study, we characterize various *B. mallei* isolates from both events (the 2004 UAE outbreak and the 2010–'11 Bahrain outbreak), using MLVA and next-generation whole genome sequencing. Our results provide evidence that the recent outbreak in Bahrain was caused by two different *B. mallei* strains, suggesting two independent introductions.

## Methods

### Ethics Statement

Glanders is a notifiable disease to the World Organization for Animal Health (OIE). As the official OIE reference laboratory for glanders in the Arabic region, the Central Veterinary Laboratory (CVRL) in Dubai is the officially authorized institution for glanders research, surveillance and eradication. All procedures involving animals were performed in strict accordance with the OIE guidelines for animal welfare using prescribed protocols.

An ethic commission comprising 4 veterinarians of the Central Veterinary Research Laboratory (CVRL) and a government veterinarian from the Ministry of Environment and Water of the UAE followed the Ministerial Decree No 384 of the year 2008 on the executive by-law of the Federal Law No 16 of the year 2007 concerning Animal Welfare. All experimental animals and treatment in this study were reviewed and approved by the Animal Ethic Committee of CVRL, and Ministry of Environment and Water of the UAE (Permit Number: *550353*).

Isolation of the strains, DNA extraction, and MLVA were performed as described previously (*8*). A total of nine *B. mallei* isolates, each from a different horse and one from the camel from both Bahrainian outbreak events (2010 and 2011) were analyzed along with 15 isolates from the 2004 outbreak in the UAE by applying the same high-resolution 23-marker VNTR assay used to type the strain from the camel [8]. To put the Bahrainian strains in a larger phylogeographic context, MLVA23 data from nine *B. mallei* isolates from the strain collection of the Bundeswehr Institute of Microbiology in Munich and previously published data from an additional 42 samples [9], [10] were included in the phylogenetic reconstruction (Table S1). Analysis of VNTR data was performed as previously described in Hornstra et al. [10].

**Figure 1:**
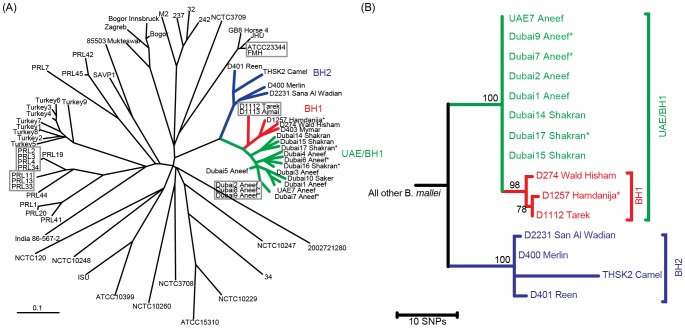
Phylogenetic relationships among isolates. **A)** Unrooted neighbor-joining tree based on 23 variable number tandem repeat (VNTR) loci demonstrating the genetic relationships of Bahrain and Dubai outbreak strains to other *B. mallei*. Colors reflect the clusters from which representatives were selected for whole genome sequencing. Asterisks indicate samples that were passaged through Guinea pigs. **B)** Whole genome SNP tree of Bahrain and Dubai genomes shows evolutionary relationships among outbreak isolates. A whole genome SNP tree including previously published genomes (not shown) confirms that the Bahrain and Dubai strains form a monophyletic group; this tree was rooted with the ATCC 23344 genome.

To assess phylogenetic relationships we sequenced and compared eight genomes from the outbreak in the United Arab Emirates from 2004 to seven genomes from the Bahrain outbreak. Using ATCC 23344 genome as a reference, we identified homologous regions using MUMmer [11], and found single nucleotide polymorphisms (SNPs) using SolSNP ((http://sourceforge.net/projects/solsnp/). After eliminating potential paralogs and positions with missing or ambiguous data among isolates, raw reads containing SNPs were aligned to the reference and inspected to verify allele calls and eliminate mixed alleles (when the minor allele frequency was greater than 10%). The final dataset was composed of 242 SNPs of which only 44 were variable among the Bahrain and Dubai genomes. Phylogenetic reconstruction was achieved using the maximum parsimony method implemented in MEGA 5.2.2 (Figure 1, panel B). The consistency index was 1.0 for all sites, indicating that the final dataset is devoid of homoplasy and that all sites are in agreement with the topology. For datasets with little homoplasy, the consistency index is a more appropriate and direct measure of accuracy than bootstrapping, however 500 bootstrap replicates revealed that the clades of interest clustered together in greater than 98% of replicate trees.

## Results/Discussion

MLVA revealed that the nine strains from the 2010–'11 outbreak in Bahrain formed two clearly separated clusters (BH1 and BH2), consisting of five and four strains each (Figure 1, panel A). Whereas BH1 was closest to the cluster consisting of various strains from the 2004 outbreak in the UAE (Figure 1, panel A), BH2, which also contained the strain from the camel sample, differed at eight VNTR markers from the closest strain (D403, Mymar) of the BH1 cluster. This suggests at least two different *B. mallei* populations were involved in the outbreak. Strains from 2010 and 2011 were found in both clusters, suggesting an outbreak that persisted across both years and was caused by two independent but temporally simultaneous introductions. The whole genome SNP phylogeny (Figure 1, panel B) confirmed the MLVA data and lends further support to the hypothesis that two different populations of *B. mallei* caused the outbreak in Bahrain.

Our results demonstrate that MLVA provides an important and useful tool for rapid initial estimations of epidemiological links among *B. mallei*. Moreover, the data suggest that MLVA can be used to study genetic diversity among *B. mallei* clones from a single outbreak. In this study, both MLVA and SNP methods revealed the same phylogenetic patterns among the three main groups (UAE/BH1, BH1, and BH2) suggesting the involvement of two genetically closely related but distinct *B. mallei* populations during the outbreak. The close relationship of the Dubai/UAE *B. mallei* population to the BH1 and BH2 populations may be indicative of a similar geographic source that has yet to be identified. Animal importation records suggest Syria and Kuwait as possible sources. This is strongly supported by the fact that one of the necropsied horses in Bahrain quarantine came directly from Kuwait.

Outbreak dynamics and natural genetic variability of *B. mallei* are not well understood due to the rarity of this disease. This outbreak provided a unique opportunity to understand outbreak dynamics as they occurred in a region that was previously free of *B. mallei*, and well monitored with records of animal importations. The failure of initial eradication efforts in 2010 is evidence for the need for continued surveillance and abatement measures even after all animal cases appear to be cleared. All animals imported from known or potentially endemic regions should be routinely tested for glanders prior to importation. Repeated testing during quarantine is also recommended as infected animals may be asymptomatic and serologically negative.

## Supporting Information

Table S1 Summary of epidemiologic and 23-locus VNTR data for 24 isolates of *Burkholderia mallei* from glanders outbreaks in UAE, Bahrain, and 42 previously published isolates.
(XLSX)
